# Factors influencing research engagement: research interest, confidence and experience in an Australian speech-language pathology workforce

**DOI:** 10.1186/1472-6963-13-144

**Published:** 2013-04-19

**Authors:** Emma Finch, Petrea Cornwell, Elizabeth C Ward, Steven M McPhail

**Affiliations:** 1 Speech Pathology Department, Princess Alexandra Hospital, Brisbane, Australia; 2 Division of Speech Pathology, The University of Queensland, Brisbane, Australia; 3 Centre for Functioning and Health Research, Queensland Health, Brisbane, Australia; 4 Behavioural Basis of Health, Griffith Health Institute, Griffith University, Brisbane, Australia; 5 Metro North Health Service District, Queensland Health, Brisbane, Australia; 6 School of Public Health & Social Work and Institute of Health and Biomedical Innovation, Queensland University of Technology, Brisbane, Australia

## Abstract

**Background:**

Recent initiatives within an Australia public healthcare service have seen a focus on increasing the research capacity of their workforce. One of the key initiatives involves encouraging clinicians to be research generators rather than solely research consumers. As a result, baseline data of current research capacity are essential to determine whether initiatives encouraging clinicians to undertake research have been effective. Speech pathologists have previously been shown to be interested in conducting research within their clinical role; therefore they are well positioned to benefit from such initiatives. The present study examined the current research interest, confidence and experience of speech language pathologists (SLPs) in a public healthcare workforce, as well as factors that predicted clinician research engagement.

**Methods:**

Data were collected via an online survey emailed to an estimated 330 SLPs working within Queensland, Australia. The survey consisted of 30 questions relating to current levels of interest, confidence and experience performing specific research tasks, as well as how frequently SLPs had performed these tasks in the last 5 years.

**Results:**

Although 158 SLPs responded to the survey, complete data were available for only 137. Respondents were more confident and experienced with basic research tasks (e.g., finding literature) and less confident and experienced with complex research tasks (e.g., analysing and interpreting results, publishing results). For most tasks, SLPs displayed higher levels of interest in the task than confidence and experience. Research engagement was predicted by highest qualification obtained, current job classification level and overall interest in research.

**Conclusions:**

Respondents generally reported levels of interest in research higher than their confidence and experience, with many respondents reporting limited experience in most research tasks. Therefore SLPs have potential to benefit from research capacity building activities to increase their research skills in order to meet organisational research engagement objectives. However, these findings must be interpreted with the caveats that a relatively low response rate occurred and participants were recruited from a single state-wide health service, and therefore may not be representative of the wider SLP workforce.

## Background

In the Australian healthcare setting, recognition of the value of research conducted in health services has stimulated interest in research capacity building for clinical health professionals [1,2]. There is now an expectation that allied health professionals will participate in evidence-based practice (EBP) and research related activities [3]. Acknowledging this, research engagement is now included in the job descriptions of clinical positions within some health organisations [4]. The organisation responsible for public healthcare service in the state of Queensland, Australia, has introduced a series of research-focused initiatives to help drive improvements in health outcomes, outlined in their 2020 Health and Medical Research and Development Strategy [5]. The initiatives focus on integrating research and health service delivery, and transferring research into clinical practice by introducing dedicated research staff positions, the provision of clinical research project grants, and staff research training [5]. The increasing focus on research has led to the implementation of programs and centres designed to stimulate research capacity building [6]. Integral to the scheme is the idea that clinical staff should receive research training to assist them to critically evaluate and apply new developments to their clinical practice [6].

Research capacity initiatives such as those introduced in Queensland are to be applauded; however they confirm only that there is an expectation for health professionals to be engaged in research activities. The extent to which Australian allied health professionals are actually interested, involved and undertaking research activities is largely unknown. The limited research in this field to date has tended to include multiple health professions in each study leading to a small number of individuals representing each profession [7,8], rather than focusing on any one allied health profession in detail. This lack of specific knowledge about the current strengths and weaknesses relating to research engagement within the allied health workforce limits our understanding of what is needed to foster research capacity amongst allied health professions. The scarcity of empirical data to inform research capacity building initiatives is also problematic for organisations committing resources to this endeavour. Furthermore, without baseline measurement of research capacity prior to the implementation of research capacity initiatives, any potential increases in research activity and engagement within a specific workforce are unable to be quantified.

Stephens and colleagues [3] surveyed the research experience of 132 Australian allied health professionals and found that overall health professionals rated themselves as having little research experience. A study by Reid and colleagues [1] similarly found that most primary healthcare workers surveyed reported having ‘little’ to ‘moderate’ research experience. In both studies, the areas with greatest research experience related to performing basic research tasks (e.g., searching for literature) with few individuals involved in publishing research [1,3]. It was noted however that respondents in both the Reid et al. [1] and Stephens et al. [3] studies indicated they had higher levels of interest than experience in research tasks. Of particular note, Stephens and colleagues [3] reported that the small number of speech-language pathologists (SLPs) (N = 15) who participated in their survey reported higher levels of interest in research than other allied health professions [3]. How this higher level of interest translates into engagement in research is unknown, particularly in relation to SLPs’ experiences conducting research or their confidence with conducting research-related activities. To date there is only a small body of literature based on empirical data pertaining to the level of research engagement of clinical SLPs.

Findings from empirical studies among SLPs indicate that despite showing positive interest in research and research related activities, very few clinicians are actively engaged in research [3]. Similarly SLPs generally have positive attitudes towards the clinical implementation of EBP [9,10], though critical appraisal of the research evidence (the intersection between EBP and research) is reported to be under-utilised by SLPs [10,11]. Studies have found only partial application of EBP principles, with SLPs tending to rely on their clinical experience and the opinions of colleagues when making clinical decisions with limited utilisation of published literature [10,11].

Although there is a scarcity of empirical data investigating SLP participation in research generating activities, there has been preliminary research into factors influencing allied health participation in EBP activities. Studies have found that participation in EBP may be associated with years of clinical experience and highest qualification obtained [12,13]. Interestingly though, this relationship is unexpectedly inverse, with less clinical experience associated with greater EBP participation [12,13]. Jette et al. [12] suggested that the association may reflect an increasing focus on EBP in contemporary education programs. Other research has found that while less clinical experience may be associated with greater confidence with the EBP process, this does not always translate into greater EBP participation [14]. It has also been proposed that research-based higher degrees (e.g., masters and doctorates) and exposure to research or EBP during a clinical fellowship year may be associated with increased EBP participation and confidence [10,12,14-16].

Popular perception suggests that the nature of the employment setting may also potentially influence research engagement, with rural health professionals often perceived to be engaged in fewer research activities than their metropolitan counterparts. McCluskey [15] compared EBP participation between metropolitan and rural occupational therapists to examine the hypothesis that metropolitan clinicians are more likely to have greater EBP knowledge and skills, and experience fewer barriers to EBP participation. However, the study failed to uphold the hypothesis with findings showing that there were no significant differences in EBP knowledge and skills between rural and metropolitan occupational therapists. Personal factors, such as level of interest in research have also been proposed to be a potential influencing factor in research engagement. For example, Stephens et al. [3] found a moderate correlation between research interest and experience, with the 15% of clinicians who undertook more research also reporting higher levels of interest in research.

At present, the research capabilities and interests of Australian SLPs beyond the use of EBP are largely unknown. The lack of knowledge about the specific research strengths and weaknesses within the SLP workforce hinders the development and delivery of appropriate research support for this workforce. Furthermore, as the factors that influence research engagement by the SLP workforce are also yet to be fully determined, there is little information to guide strategies for research capacity building and workforce development. In order to better inform research training and engagement for SLPs, the aim of the present study was to investigate the current research interest, confidence and experience in the SLP healthcare workforce, and factors that predict research engagement.

## Methods

### Design

A cross-sectional design using a customised web-based survey was undertaken.

### Participants

The survey target group included practising SLPs working within the organisation providing public healthcare services for the state of Queensland, Australia [17]. This organisation is the largest employer of SLPs in this state [18]. There were no specific exclusion criteria. The SLPs were sourced through the Leaders in Speech Pathology group, whose members include the department directors from all SLP services within the organisation, as well as university and key industry representatives. Department directors within the Leaders in Speech Pathology group were asked to distribute the survey link to their practising SLP staff via staff email lists. Based on position data available from this group at the time of the survey, there were approximately 330 SLPs working in the organisation (including full-time and part-time staff). This figure was taken as the total number of recipients who would be given access to the survey link.

### Measures

**Figure 1:**
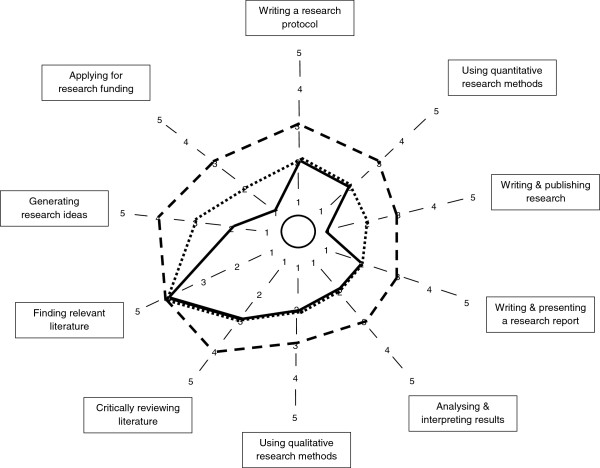
**Median self-rated research experience, confidence and interest.** Note. Research experience (solid line), Research confidence (small dots), Research interest (dash). 1 = None; 5 = Very.

The customised survey consisted of four key sections: consent, demographic information, adaptation of the Research spider tool [19], and additional research participation questions. The first section of the survey involved an online information sheet and standard consent questions. Respondents were able to progress to the remainder of the survey only if they answered ‘yes’ to the consent questions. The second section of the survey consisted of demographic questions (e.g., years of experience, geographical location, the nature of primary caseload) reported in multiple choice format. The third section of the survey was based on the Research spider tool [19]. The ‘Research spider’ is a star-plot questionnaire designed for health professionals to self-rate their level of experience from ‘none’ through to ‘very’ experienced on 10 specific research tasks (e.g., publishing research, analysing and interpreting results; see Figure 1) [19]. The ‘Research spider’ has performed well on measures of reliability and validity, with Smith et al. [19] reporting high correlations between individuals’ mean scores on the spider and their actual research experience (Spearman’s rank correlation = −.73). The authors also reported excellent test-retest reliability (Spearman’s rank correlation = .95) [19]. In the current study, additional questions were added to the ‘Research spider’ tool to explore self-ratings of confidence and interest across the 10 research tasks. Respondents rated their experience, confidence and interest on the tasks on a scale from 1 to 5 with 1 = none, 2 = little, 3 = some, 4 = moderate, and 5 = very. Respondents were also required to rate their overall research experience, interest and confidence, and the degree to which they thought research had the potential to influence their clinical practice or the way their team provides their services. The final section of the survey asked respondents how many times they had completed each of the 10 research tasks from the ‘Research spider’ over the last 5 years.

### Procedures

Ethical approval for the study was obtained from the University of Queensland Human Research Ethics Committee with gatekeeper approval from the Queensland Health Leaders in Speech Pathology group. Members of the Leaders in Speech Pathology group agreed to distribute the anonymous survey to all employed SLPs in their services. The link to the secure web-based survey was provided to all leaders for forwarding to their staff. The survey remained open from 25 May 2011 until 5 August 2011. Reminder emails were sent three times to encourage participation.

### Data analysis

**Figure 2:**
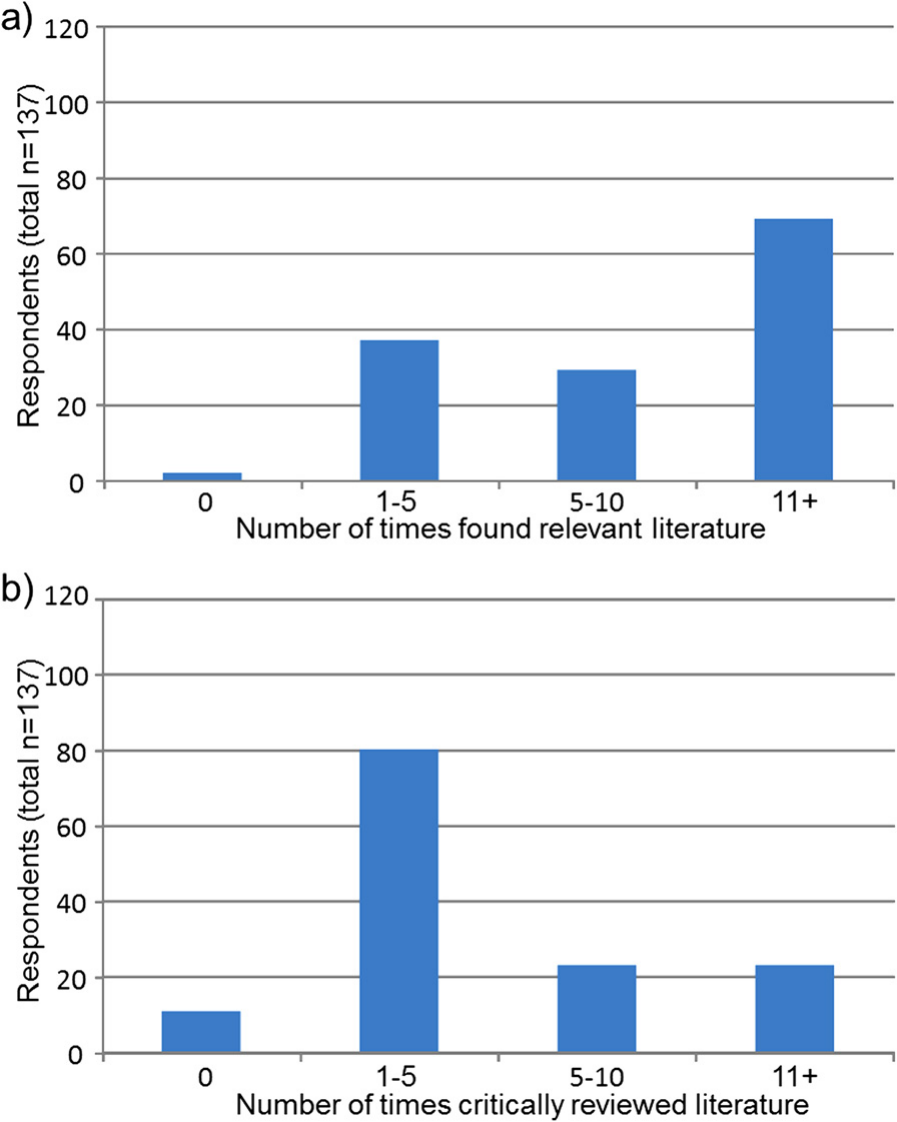
Number times respondents a) found relevant literature and b) critically appraised literature within past 5 years.

**Figure 3:**
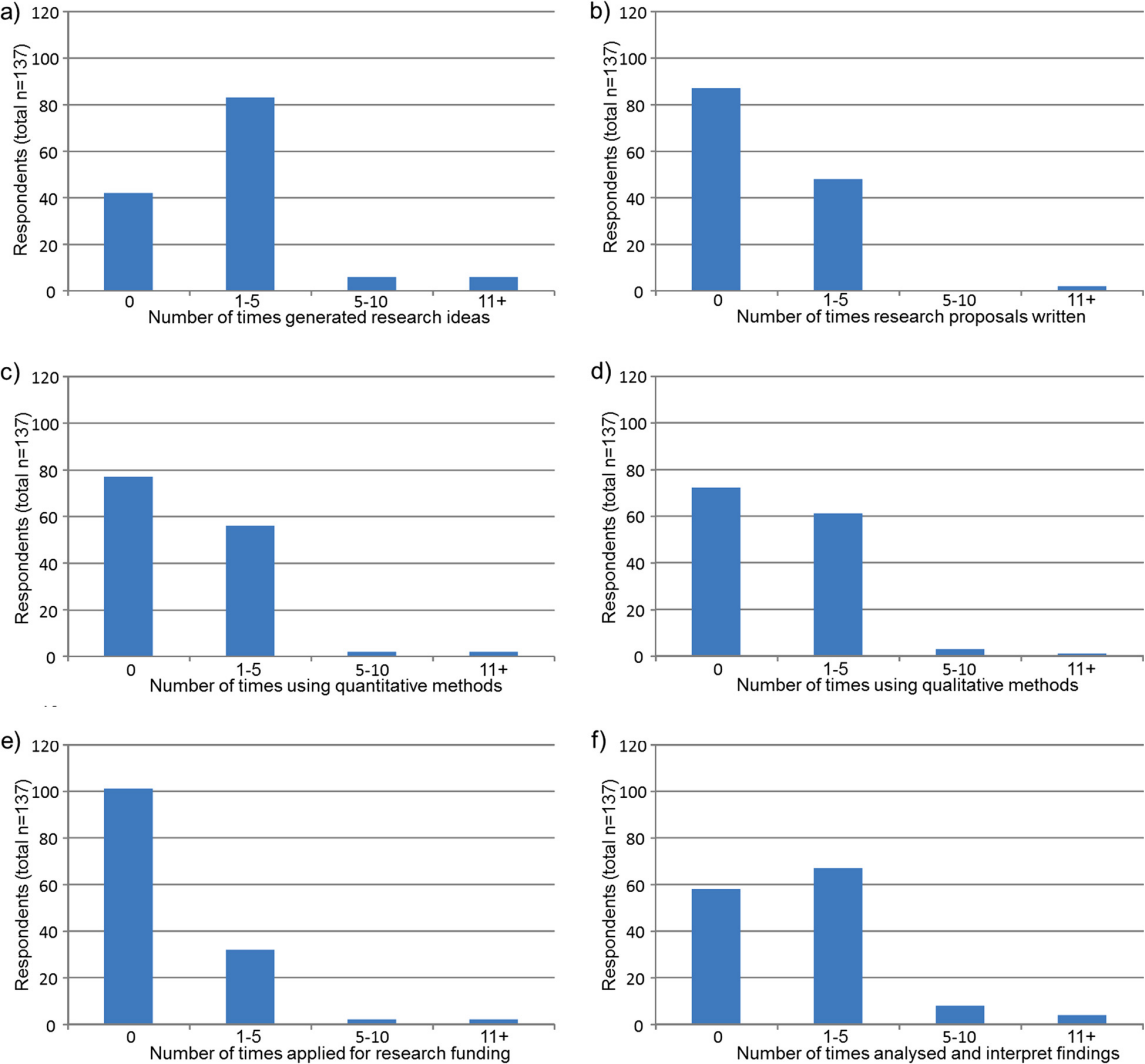
Number of times respondents a) generated research ideas, b) wrote proposals, c) used quantitative or d) qualitative methods, e) wrote research proposals and f) analysed findings within past 5 years.

**Figure 4:**
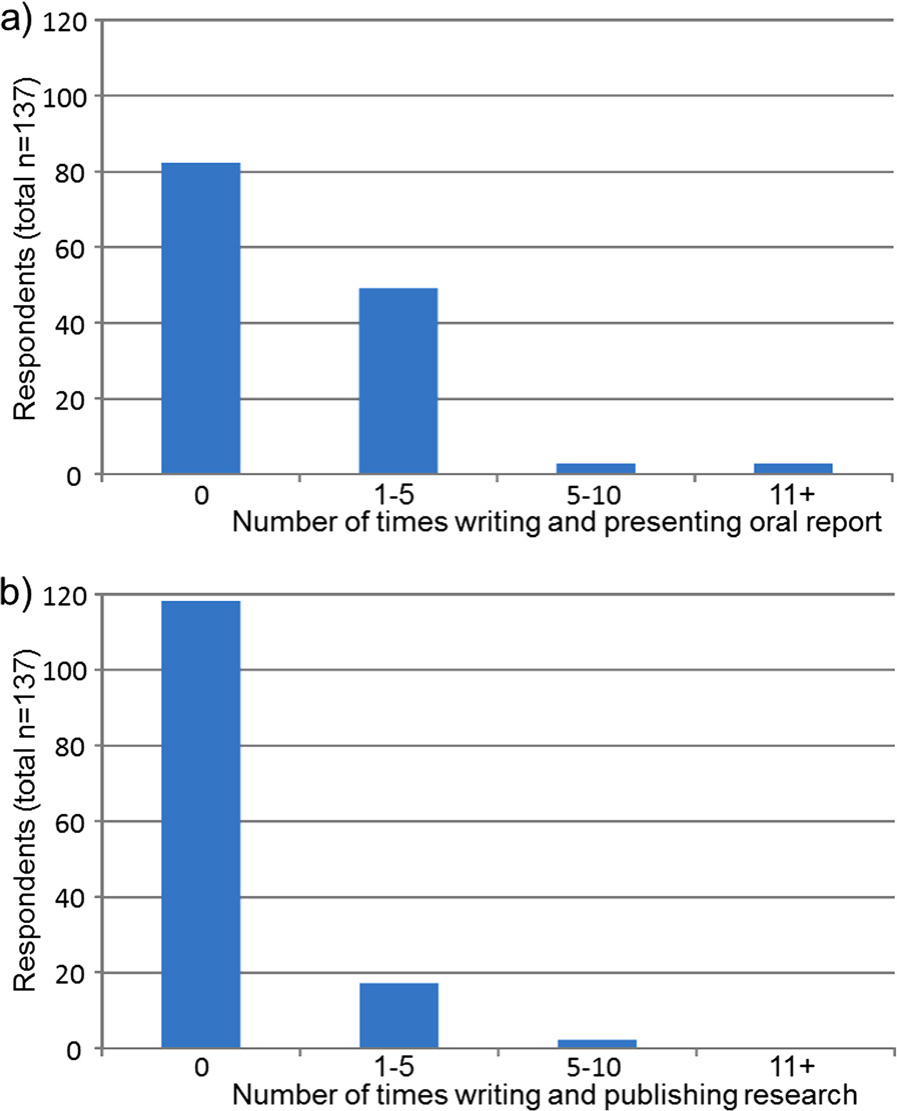
Number of times respondents a) writing and presenting oral report and b) writing and publishing research within past 5 years.

Prior to data analysis, participants’ geographical locations were classified as ‘metropolitan’ or ‘non-metropolitan’ based on health service district classifications for ease of reporting and small respondent numbers in some regional districts. Descriptive statistics were used to outline participant demographic information (Table 1) and analyse the survey data pertaining to ratings of interest, experience and confidence on each of the 10 research tasks (Table 2). Responses to the research spider tool were presented graphically in the corresponding star-plot (Figure 1). Reports of the number of times participants reported completing the 10 tasks listed in the research spider tool were presented in frequency histograms (Figures 2, 3 and 4). This included two tasks pertaining to finding and critically appraising literature (Figure 2), six tasks related to planning and conducting research (Figure 3) and two tasks dealing with disseminating research findings (Figure 4). Multiple regression using Predictive Analytics Software (PASW, version 18) (2009) with an enter method (that is all of the independent variables included in the one model [20]) was used to examine the predictive value of: years of clinical experience; geographical location; highest qualification obtained; current position classification level; and overall interest in research (independent variables), on the total number of research related tasks performed (dependent variable). For this regression, the sum of each participant’s report of the number research tasks completed was used as the dependent variable. An alpha level of .05 was used for all analyses. Prior to the multiple regression analysis, multicollinearity (a potential confounder in multiple regression analyses) was examined (using correlational analyses), but no strong associations between independent variables existed.

**Table 1:** Participant demographic information

	**n**	**(%)**
**Gender**		
Female	134	(98%)
Male	3	(2%)
**Highest qualification**		
Bachelor degree	89	(65%)
Bachelor degree with honours	30	(22%)
Coursework masters	13	(9%)
Research masters/Doctorate	5	(4%)
**Years of clinical experience**		
Less than 5	53	(39%)
5 – 10	34	(25%)
Greater than 10	50	(36%)
**Employment location**		
Metropolitan	75	(55%)
Non-metropolitan (rural and regional)	62	(45%)
**Current position classification level**		
HP 3	53	(39%)
HP 4	52	(38%)
HP 5	25	(18%)
HP 6	6	(4%)
HP 7	1	(1%)
**Current position type**		
Clinical	118	(86%)
Management	12	(9%)
Clinical education	5	(4%)
Research	2	(1%)
**Current caseload majority**		
Adult	77	(56%)
Neonates	1	(1%)
Paediatrics	42	(31%)
Adolescents	5	(4%)
Mixed adult/paediatric	12	(9%)
**Employment type**		
Full-time	99	(72%)
Part-time	38	(28%)
**Employment status**		
Permanent	85	(62%)
Locum/temporary	52	(38%)

*Note.* HP3 represents an entry-level, base grade clinician while the higher HP levels represent progressively higher expertise and influence.

**Table 2:** Interest, experience or confidence on the 10 Research spider tasks according to percentage of respondents

	**Task**	**Respondent ratings n (%)**				
		**None**	**Little**	**Some**	**Moderate**	**Very**
Interest	Finding relevant literature	0 (0%)	3 (2%)	15 (11%)	56 (41%)	63 (46%)
	Critically reviewing literature	1 (1%)	11 (8%)	39 (28%)	42 (31%)	44 (32%)
	Generating research ideas	0 (0%)	18 (13%)	44 (32%)	38 (28%)	37 (27%)
	Writing a research proposal	14 (10%)	25 (18%)	48 (35%)	29 (21%)	21 (15%)
	Using quantitative research methods	7 (5%)	29 (21%)	49 (36%)	32 (23%)	20 (15%)
	Using qualitative research methods	6 (4%)	27 (20%)	46 (34%)	37 (27%)	21 (15%)
	Applying for research funding	19 (14%)	28 (20%)	38 (28%)	26 (19%)	26 (19%)
	Analysing and interpreting results	5 (4%)	22 (16%)	47 (34%)	36 (26%)	27 (20%)
	Writing and presenting an oral report	14 (10%)	28 (20%)	37 (27%)	35 (26%)	23 (17%)
	Writing and publishing research	18 (13%)	26 (19%)	35 (26%)	29 (21%)	29 (21%)
Experience	Finding relevant literature	0 (0%)	6 (4%)	46 (34%)	68 (50%)	17 (12%)
	Critically reviewing literature	1 (1%)	30 (22%)	55 (40%)	41 (30%)	10 (7%)
	Generating research ideas	19 (14%)	50 (36%)	41 (30%)	20 (15%)	7 (5%)
	Writing a research proposal	59 (43%)	36 (26%)	30 (22%)	12 (9%)	0 (0%)
	Using quantitative research methods	32 (23%)	51 (37%)	40 (29%)	14 (10%)	0 (0%)
	Using qualitative research methods	28 (20%)	54 (39%)	36 (26%)	17 (12%)	2 (1%)
	Applying for research funding	83 (61%)	26 (19%)	19 (14%)	9 (7%)	0 (0%)
	Analysing and interpreting results	28 (20%)	45 (33%)	44 (32%)	19 (14%)	1 (1%)
	Writing and presenting an oral report	51 (37%)	34 (25%)	30 (22%)	18 (13%)	4 (3%)
	Writing and publishing research	75 (55%)	30 (22%)	22 (16%)	10 (7%)	0 (0%)
Confidence	Finding relevant literature	0 (0%)	13 (9%)	54 (39%)	55 (40%)	15 (11%)
	Critically reviewing literature	2 (1%)	33 (24%)	55 (40%)	37 (27%)	10 (7%)
	Generating research ideas	12 (9%)	54 (39%)	42 (31%)	23 (17%)	6 (4%)
	Writing a research proposal	48 (35%)	50 (36%)	27 (20%)	11 (8%)	1 (1%)
	Using quantitative research methods	37 (27%)	52 (38%)	34 (25%)	13 (9%)	1 (1%)
	Using qualitative research methods	30 (22%)	53 (39%)	35 (26%)	15 (11%)	4 (3%)
	Applying for research funding	63 (46%)	45 (33%)	21 (15%)	8 (6%)	0 (0%)
	Analysing and interpreting results	28 (20%)	46 (34%)	45 (33%)	15 (11%)	3 (2%)
	Writing and presenting an oral report	42 (31%)	38 (28%)	35 (26%)	19 (14%)	3 (2%)
	Writing and publishing research	59 (43%)	41 (30%)	26 (19%)	10 (7%)	1 (1%)

## Results

### Respondent demographic information

A total of 158 SLPs responded to the survey; however, due to incomplete responses only 137 responses were included in the statistical analyses, representing an estimated response rate of 42% (137/330). This response rate is higher than anticipated based on previous research, which has indicated that the median response rate to survey data of this nature to be 26%. [21]. Demographic information for the 137 complete respondents is displayed in Table 1. The sample was predominantly female with a bachelor degree as the highest qualification. Most respondents were employed permanently in a full-time clinical position with a slight preponderance of adult caseloads. The majority of respondents were currently employed at the first (HP3) or second (HP4) industrial classification level for allied health staff, with the higher HP levels representing progressively higher expertise and influence [4]. A slightly higher number of respondents were employed in metropolitan settings compared with non-metropolitan settings. Similar percentages of respondents had less than 5 years clinical experience or greater than 10 years of clinical experience (39% and 37% respectively) (see Table 1).

### Research interest, experience and confidence

Figure 1 provides a summary of the levels of interest, experience and confidence for SLPs on each research task. When asked to rate their overall research interest, experience and confidence (i.e., rating their research interest, experience and confidence in general rather than with respect to individual research tasks), respondents reported moderate levels of interest (Median interest = 4, ‘moderate’ interest), but only low levels of experience and confidence (Median experience = 2 ‘little’ experience, Median confidence = 2, ‘little’ confidence). When rating their interest, confidence and experience on the 10 individual research tasks, respondents’ levels of interest in all 10 research tasks ranged from only ‘some’ to ‘moderate’ interest (see Figure 1). For all tasks except finding relevant literature, SLPs reported higher levels of interest than experience and confidence. The task with the greatest experience level was finding relevant literature. This was also the only item where interest, experience and confidence were ranked equally. For all other research activities, SLPs displayed low levels of experience. In 7 of the 10 research tasks, SLPs reported the same level of experience as confidence for the research tasks. The remaining three tasks: generating research ideas, applying for research funding, and writing and publishing research saw respondents report higher levels of confidence than experience (Figure 1).

In addition to the star plot, frequency (and percentage) of responses was tabulated for levels of interest, experience or confidence on the 10 research tasks (Table 2). The research tasks of most interest were finding and critically reviewing literature, and generating research ideas. Conversely, research related tasks that SLPs were least interested in involved applying for research funding, writing and presenting an oral report, and writing for publication. A similar pattern of responses was present for both experience and confidence in completing the ten research tasks, with more experience and confidence in finding and critically appraising research literature as well as generating research ideas (Table 2). While at the other end of the spectrum, respondents infrequently considered themselves to be experienced or confident with applying for research funding, writing and presenting an oral report, and writing for publication (Table 2).

The pattern of responses for reports of research tasks undertaken within the past 5 years (Figures 2, 3 and 4) was commensurate with interest, experience and confidence reported in Table two. Tasks related to finding and critically appraising literature (Figure 2) were more frequently undertaken than tasks related to planning, or undertaking research (Figure 3) or disseminating research through presenting a research paper or writing and publishing research (Figure 4). Despite literature searching being the most frequently undertaken research task, only 69 (50%) respondents reported that they had conducted 11 or more literature searches over the last five years (Figure 2). Thirty-nine (28%) respondents reported they had found relevant literature on no more than five times occasions in the past five years. Fewer respondents reported completing tasks related to planning and conducting research projects (Figure 3) or disseminating study findings (Figure 4) than reviewing literature. Six tasks had not been completed by the majority of respondents including having written a research proposal, used quantitative research methods, used qualitative research methods, applied for research funding, presented a research paper or published a research paper (Figures 3 and 4).

### Factors predicting engagement in research

Multicollinearity analysis for the independent variables in the model revealed only non-significant or weak associations (rho<0.25) between each independent variable combination with one exception [22]. This exception was the moderate correlation between years of experience and current position classification level (rho = .675) [22]. However, these two variables were still entered into the regression model, as the association was not strong [22] and years of experience do not always equate with position classification within organisations that deliver health services [4]. The results of the multiple regression analysis predicting engagement in research are presented in Table 3. The regression model was significant (r-squared = 0.368, p<0.001), indicating that the model had predictive ability in identifying the degree to which SLPs were engaged in research. The independent variables that significantly predicted engagement in research were highest qualification obtained (p <.001), current position classification level (p = .037) and overall interest in research (p = .026) (Table 3). Years of clinical experience and geographical location did not significantly predict engagement in research.

**Table 3:** Linear regression model (r-squared = 0.368, p<0.001) results for predicting total number of research tasks undertaken

**Predictor**	***B***	***SE***	***95% CI for B***		***t***	***p***
			***Lower***	***Upper***		
(Constant)	9.834	1.695	6.481	13.187	5.803	.000
Qualification	3.170	.525	2.131	4.208	6.038	.000*
Years of clinical experience	.172	.658	−1.130	1.473	.261	.794
Location	−1.309	.862	−3.015	.397	−1.517	.132
Overall interest in research	.858	.382	.103	1.614	2.247	.026*
Current position level	1.344	.636	.086	2.602	2.113	.037*

*Note. B*=Beta, *SE* = Standard error; *CI*= Confidence Intervals, *t*= t-statistic, *p* = *p*-value (significance); * = *p* < .05.

## Discussion

The current investigation indicated that SLPs within this workforce had moderate levels of interest in participating in research activities. However, their experience and confidence in completing research tasks other than finding and reviewing literature was limited. Respondents did not frequently undertake the majority of the ten research tasks. Engagement in research activities was predicted by highest qualification obtained, current job classification level and overall interest in research. These predictors of research engagement may offer a useful starting point for strategic initiatives intended to increase the level of research engagement amongst SLP workforce. Specifically, this may include strategies to foster the attainment of research-related qualifications and promote general interest in research among individual clinicians or groups of clinicians.

Variation in the level of research interest existed across the ten research tasks. The median level of interest was generally either ‘some’ or ‘moderate’ (i.e., median ratings ranged from 3 to 4 on the 5 point interest rating scale) for most tasks. While interest was greatest in the tasks related to finding literature, appraising literature and generating research ideas, it was encouraging that a portion of respondents did report being very interested in each of the other seven tasks. Less encouraging, was the greater proportion of respondents who indicated little or no interest in these seven remaining research related tasks. While this study has provided empirical evidence indicating that an association between research interest and research engagement exists, a salient point for future investigations is the nature of causality of this link between interest and engagement.

The finding from this investigation that research interest was associated with engagement has face validity and is consistent with prior research on the topic of research engagement. Stephens and colleagues [3] found a moderate correlation between interest in research and research experience, and observed that higher interest levels were associated with greater research engagement. Hence stimulating clinician interest in research activities would appear to be an integral step in enhancing research engagement. To this end, the proportion of respondents who reported having a moderate to high level of interest in partaking in more advanced research activities may be the individuals most likely to respond favourably to initiatives designed to stimulate research activity. However, it is also plausible that SLPs who were exposed to research through participation in a structured research activity subsequently developed a stronger interest in research related tasks. Regarding the proportion of individuals reporting no interest, further qualitative research is warranted to determine the barriers or other issues influencing this opinion, and identify potential facilitators that may foster an interest (and engagement) in research.

The findings from this research are consistent with previous literature that indicated the majority of allied health professionals have limited experience conducting research related tasks. In previous studies, moderate to high levels of experience with research tasks were found in only 2 or 3 areas covered by the research spider tool [1,3]. As observed by both Reid et al. [1] and Stephens et al. [3] in their populations, the area of greatest experience in the SLP cohort was finding relevant literature. Searching for, and appraising, research literature may be considered one of the more rudimentary research tasks, but also represents a significant aspect of EBP and is the initial step in ensuring that clinical practice is driven by evidence. This promising data indicates that many clinicians are indeed participating in a task that is central to both EBP and research generating activities. In some clinical settings, systematic training in conducting literature searches as well as the formation of journal clubs and EBP groups has helped to train the clinical workforce on how to conduct literature searches to answer clinical questions [23,24]. Searching for literature is also an integral skill developed in undergraduate training programs for all students. Hence it is likely that the relative strength observed in levels of interest, engagement and confidence in the searching for literature task appears to reflect activities in an area that is perceived as having direct relevance to the clinicians' daily activities. Furthermore, literature searching is a process in which most SLPs have historically received training or have support for in the workplace.

One concerning finding from the present study was that 69 (50%) of the respondents had completed two or less literature searches per year over the previous 5 years despite this task being reported as having the highest level of interest, experience and confidence. Furthermore 39 (28%) of respondents had searched for literature less than once per year (on average) over this time. While the current study did not explore reasons for low levels of research activity, previous research has suggested that SLPs often use the opinions of colleagues or their own clinical judgement when making clinical decisions, rather than searching published journal articles [10,11]. This may also be the case in the current cohort. Further investigation of this issue is warranted to determine whether or not the low frequency of literature searching reflects (a) a need for further support and training in the components of literature searching and critical appraisal, (b) an issue with availability of resources (e.g., lack of access to academic databases and online journals), (c) a need for further ongoing initiatives designed at increasing the frequency with which an interest in finding and appraising literature is translated into an actual literature search (and appraisal) being undertaken as a part of routine practice or (d) a more complex discrepancy between self-reported survey behaviour and actual activities undertaken in real-life daily practice.

Few SLPs had completed more complex research tasks including applying for research funding, writing and publishing a research paper. This was also consistent with prior research in other related populations [1,3]. In general terms, SLPs in this investigation reported having little or no confidence in their ability to undertake the more specialised research tasks. For example, only 8 (6%) and 11 (8%) respondents reported moderate or high levels of confidence in applying for research funding or writing and publishing a research paper (respectively). Similarly, few participants had frequently performed the more complex or specialised research activities (Figures 3 and 4) over the past five years.

Findings from this study indicate that formal research capacity building strategies are likely to be required to engage allied health staff in more complex research tasks beyond literature searching and review. There are a number of strategies that have potential to address this low level of research activity in order to achieve organisational aims of increased research capacity within the healthcare workforce. These strategies may include training workshops (with allied health relevant interactive activities) [25], mentoring by colleagues who are experienced in undertaking clinical research [2,26], and active recruitment of SLPs to undertake research higher degrees [12,14,15]. This latter strategy is particularly pertinent given that in the present study, highest qualification obtained was a significant predictor of research engagement. The finding is not surprising given that many postgraduate qualifications (e.g., masters and doctorates) are research-focused, so individuals with these research-based higher degrees would be expected to have well-developed research skills. Indeed, previous research with occupational therapists and physiotherapists reported that individuals with research higher degrees are more likely to be able to generate clinical research questions, search databases and understand research terminology, and be more confident undertaking these tasks [12,14,15].

In addition to level of academic training, a higher position classification level was also found to predict research engagement in the current cohort. A number of factors are likely to have contributed to this finding. It is customary for senior clinical positions to have research activity built into the position description [4]. Hence there is recognition of the importance of demonstrating research engagement by those individuals serving in more senior roles. Individuals in more senior roles are also often seen as clinical leaders in their fields and therefore may have more opportunity to become involved in university led research activities than clinicians in more junior positions.

Contrary to popular perception, geographical location was not a predictor of research engagement [25,26]. Findings from this investigation indicated that a SLP worked in a metropolitan setting or a non-metropolitan or rural setting did not impact upon their research engagement. This finding is concordant with previous empirical EBP research, which found no difference in EBP skills between city and rural occupational therapists [15]. To ensure that this positive finding of equality across metropolitan and regional or rural services remains, it is important that future research capacity building opportunities and strategies are made equally accessible to non-metropolitan clinicians. Recent technological advances such as videoteleconferencing could be used to facilitate this process.

### Limitations and future directions

Although the authors acknowledge that the small sample and the low response rate of the current study may limit the generalisation of the results, it is noted that the sample demographics were not dissimilar to the total SLP workforce demographics released by the Speech Pathology Registration Board of Queensland [18], supporting the potential representative nature of the current sample. Notably, while the relatively high proportion of clinicians in the lower two industrial position classification (HP3 and HP4) may have potentially influenced the study results toward lower levels of research engagement, this preponderance of HP3 and HP4 positions is representative of the typical SLP workforce in the state. However, the sample was limited to only SLPs working within the organisation responsible for public healthcare service in the state Queensland, Australia. SLPs from other health services may not have responded in the same way as participants in this investigation. Further research among SLPs from other organisations, including those based in education and private practice, as well as from other geographical locations is warranted. Future research could also explore the factors influencing research engagement further through individual in-depth interviews or focus group discussions in order to identify other targets for research capacity building. Similarly, exploring the reasons why certain individuals have no interest in research and no level of research engagement should be further examined to see if perceived barriers can be addressed.

## Conclusions

The results of the present study suggest that strategies and initiatives to increase the research skills and confidence of Australian SLPs are warranted in order to meet organisational research engagement objectives. Overall, the present study found that respondents generally reported higher levels of interest in research than confidence and experience, with many respondents reporting limited experience and participation on most research tasks. Research engagement was predicted by highest qualification obtained, current position classification level and overall interest in research. The current findings have identified issues that can be targeted with strategic activities to enhance research capacity building initiatives.

## Abbreviations

SLPs: Speech Language Pathologists; EBP: Evidence-Based Practice.

## Competing interests

The authors declare that they have no competing interest.

## Authors’ contributions

EF, PC and ECW were equally involved in the conceptualization and design of the study, data analysis and drafting, appraisal and editing of the manuscript. SM contributed to data analysis, interpretation and manuscript drafting, appraisal and editing. All authors read and approved the final manuscript.

## Pre-publication history

The pre-publication history for this paper can be accessed here:

http://www.biomedcentral.com/1472-6963/13/144/prepub
